# Persistent elevation in incidence of pneumonia in children in England, 2023/24

**DOI:** 10.2807/1560-7917.ES.2024.29.32.2400485

**Published:** 2024-08-08

**Authors:** Daniel Todkill, Theresa Lamagni, Richard Pebody, Mary Ramsay, Daisy Woolham, Alicia Demirjian, Antoine Salzmann, Meera Chand, Helen E Hughes, Christopher Bennett, Russell Hope, Conall H Watson, Colin S Brown, Alex J Elliot

**Affiliations:** 1 United Kingdom Health Security Agency (UKHSA), London, United Kingdom; 2 Warwick Medical School, Coventry, United Kingdom; 3 Evelina London Children’s Hospital, London, United Kingdom

**Keywords:** *Mycoplasma pneumoniae*, syndromic surveillance, epidemiology, England, bacterial infections, pneumonia

## Abstract

Since November 2023, the absolute number of attendances at emergency departments for pneumonia among children aged 5–14 years in England have been above expected levels for the time of year. This increased signal peaked during March 2024 but then persisted into early summer 2024 despite decreases in prevalence of seasonal respiratory pathogens. Record linkage between emergency department and laboratory databases points to this unusual activity being driven largely by *Mycoplasma pneumoniae*.

Seasonal respiratory infections place significant pressures on healthcare services in England during each winter. In England, the UK Health Security Agency (UKHSA) coordinates a respiratory surveillance programme that monitors and informs on the timing and burden of respiratory activity each year. Here, we report on an unusual elevation in emergency department (ED) pneumonia attendances in children aged 5–14 years during 2024, detected through the UKHSA syndromic surveillance programme and we present the investigation to determine the underlying cause of this unusual activity.

## Acute respiratory infection surveillance in England

Presentations of respiratory infections to healthcare services typically peak during the winter months, causing annual surges and pressures that can impact on acute healthcare services. Within England, the UKHSA coordinates a comprehensive and well-established respiratory surveillance programme that monitors relevant epidemiological indicators through a suite of systems. Laboratory reports, sentinel general practitioner (primary care) surveillance, real-time syndromic surveillance (including telehealth calls and ED attendances) and hospital admissions, among others, inform respiratory infection activity and the impact on the population and health system. This is observed through respiratory indicators ranging from mild, self-limiting acute respiratory presentations, e.g. telehealth calls for symptoms of common cold, influenza-like illness and acute respiratory infection (ARI) presentations, to more severe presentations including severe ARI (SARI) and pneumonia. The syndromic surveillance component of the UKHSA respiratory programme monitors all-hazard threats to public health but has a comprehensive collection of respiratory infection surveillance indicators. Calls to a National Health Service (NHS) national telephone helpline (NHS 111) [1] and an online NHS 111 symptom checker are reviewed daily in parallel with primary care consultations (from both scheduled in-hours and unscheduled out-of-hours services), ambulance dispatch calls [2] and ED attendances [3]. To ensure that healthcare services and policymakers are informed of health service pressures, enhanced seasonal respiratory surveillance is conducted by UKHSA from October through May during each respiratory infection season [4].

## Increased emergency department pneumonia attendances in children

**Figure 1:**
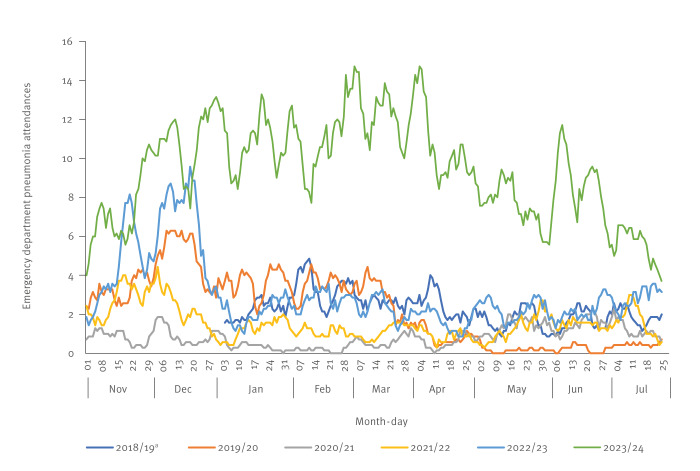
Daily emergency department attendances for pneumonia in children aged 5–14 years, England, 1 January 2019–28 July 2024 (n = 5,517)

^a^ Daily emergency department attendance data for 2018/19 were only available from 1 January 2019.

During November 2023, ED pneumonia attendances surpassed UKHSA syndromic historical exceedance thresholds indicating that activity was above expected levels. Further investigation revealed that the increase was particularly observed in children aged 5–14 years. This signal continued to exceed historical thresholds and remained at higher-than-expected levels throughout December 2023. During January 2024, the number of ED attendances would typically have been expected to fall in line with ED pneumonia attendance historical baselines, however this signal persisted at heightened levels from February to July 2024. While the number of ED attendances with a diagnosis of pneumonia in the 5–14-year age group was relatively low (2,532 attendances between 1 November 2023 and 28 July 2024) compared with older age groups (45,003 in ages ≥ 15 years during the same time period), these were markedly higher than numbers of attendances recorded during the same time period in preceding years. Excluding incomplete and pandemic years, the mean number of attendances in the 5–14-year age group during the same time period in 2019/20 and 2022/23 were 607 and 818, respectively (Figure 1).

Data from 29 February 2020 and 2024 have been removed.

From 2019 up to 28 July 2024, the ED syndromic surveillance system included 114 EDs in England, and the SNOMED-CT codes predominantly comprising the ‘pneumonia’ indicator were ‘2333604007 Pneumonia’ (67%), ‘278516003 Lobar Pneumonia’ (29.5%) and ‘385093006 Community Acquired Pneumonia’ (2.7%).

The ED pneumonia signal was further investigated by using an alternative ED attendance data feed received by UKHSA, which contained identifiable ED attendance records but with a slightly longer time lag compared to the syndromic surveillance ED data. The Emergency Care Dataset (ECDS) is a national NHS England dataset providing information on ED attendance in England [5]. A multi-stage linkage algorithm using a combination of NHS number, hospital number, date of birth, sex and full name was used to link laboratory reports from the national Second-Generation Surveillance System (SGSS) to ED attendances (ECDS) with clinical diagnosis of pneumonia (using the same SNOMED-CT codes as the Syndromic Surveillance), where attendance dates were within 7 days of the reported sample date.

**Figure 2:**
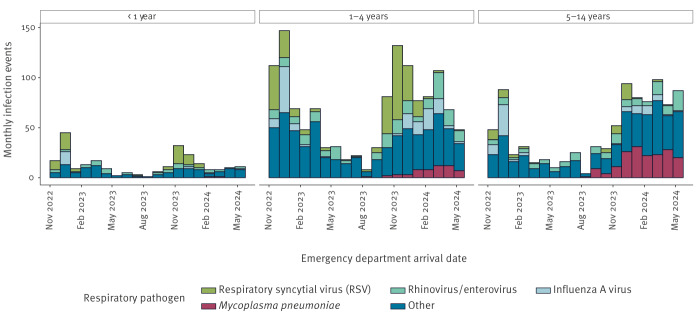
Daily trends in respiratory pathogens associated with emergency department attendances for pneumonia by age, England, 1 Nov 2022–1 May 2024 (n = 2,334)

During the period 1 November 2022–1 May 2024, 14,096 ED attendances related to pneumonia were identified in the ECDS. Of these, 2,334 (16.6%) were linked to a positive microbiology result in SGSS. The most commonly identified pathogens were RSV (16.6%; n = 387), influenza A virus (9.8%; n = 228) and *Mycoplasma pneumoniae* (MP; 9.3%; n = 216) with peaks for influenza and RSV in line with the usual seasonal/annual patterns. However, during the period between 1 January 2024 and 1 May 2024, there was an increase in the proportion of cases, primarily in the 5–14-year age group, with this pathogen being identified in 32.2% (n = 105 cases of MP among 326 linked ‘pneumonia’ attendances) of ED attendances within this age group, and a rising trend in MP in children aged 1–4 years (Figure 2).

## Discussion

In summary, here we describe a persistent elevation in the number of ED attendances amongst children aged 5–14 years with a diagnosis of pneumonia. We show that this unusual activity was driven by heightened and prolonged MP activity in England, the first MP epidemic season (winter 2023/24) following the COVID-19 pandemic. Winter respiratory infection pressures are commonly caused by a range of respiratory and gastrointestinal pathogens, including influenza virus, rhinovirus, norovirus, respiratory syncytial virus (RSV) and now severe acute respiratory syndrome coronavirus 2 (SARS-CoV-2). *Mycoplasma pneumoniae* is also a common bacterial cause of community-acquired infection in school-aged children (5–14 years), estimated to be responsible for between 8 and 40% of community-acquired pneumonia admissions to the hospital [6,7]. While the majority of MP infections are mild, presenting with non-specific upper and lower respiratory tract symptoms [8], some cases are associated with severe pneumonia, asthma exacerbation [9], extra-pulmonary manifestations and severe disease. Prevalence estimates are complicated by the fact that testing for this pathogen has changed considerably in the past few years, with a shift from limited access to serological testing to more widespread availability of PCR testing, particularly on multiplex respiratory PCR panels. This is further complicated by notable degree of asymptomatic carriage, particularly in children [10]. Of concern are increasing rates of macrolide resistance in some parts of the world [11], as this class of antibiotics has been a first line therapy of MP infections to date, particularly in children.

Cyclical epidemics of MP are typically observed every 3–5 years, with varying trends in epidemic patterns between countries, and in England every 4 years [12]. The last epidemic to affect Europe and Asia happened in late 2019 but was rapidly ended with the emergence of SARS-CoV-2 in early 2020 and the implementation of non-pharmaceutical interventions (NPIs) as part of the pandemic response. Winter 2023/24 has, however, shown to be an epidemic season for MP in England, with exceedances in MP laboratory detections observed earlier in the winter season [13]. Globally, increases in MP have also been observed in China [14], where a nationwide epidemic of MP infections has occurred since late 2023, and increases have also been reported in the Netherlands [15], Denmark [16], the United States [17] and Australia [18].

Given the 4-to-5-year MP epidemic cycle, the 2023/24 epidemic was not unexpected, however 2023/24 has been an unusually prolonged season. Of note, a similar pattern of increased ED attendances for pneumonia was not seen in syndromic ED data during the last epidemic season in 2019/20. The reasons for this are not clear, however the atypical, prolonged activity could be a result of MP being re-established following the pandemic-related NPIs. Other studies have observed a reduction in transmission, or atypical activity across a range of infectious diseases (since the previous cycle of increased MP incidence) that have been attributed to the impact of NPIs implemented during the COVID-19 pandemic [19].

Early seasonal exceedances in MP were detected during November 2023 using established laboratory reporting networks. At that time, public health action was taken in the form of issuing a briefing note alerting to MP activity, particularly aimed at paediatricians and emergency care clinicians [13]. During the winter 2023/24, the value of real-time syndromic surveillance was highlighted; syndromic signals alerted to persisting late respiratory presentations at a time when other routine seasonal pathogen activity had declined. Bespoke statistical exceedances and epidemiological interrogation of the daily syndromic signal highlighted persisting healthcare-seeking attendance for acute pneumonia, which was subsequently determined to be caused by MP. UKHSA syndromic surveillance has continued to routinely assess pneumonia attendances in EDs across England, monitoring syndromic data on a daily basis and publishing epidemiological updates each week. The most recent UKHSA ED syndromic surveillance reports (published 1 August 2024) illustrate that pneumonia ED attendances are now approaching seasonally expected levels [20].

## Conclusion

UKHSA continues to operate a multi-partite and comprehensive respiratory surveillance programme incorporating a wide range of community, syndromic and laboratory surveillance systems for monitoring known seasonal pathogens each winter. The benefit of incorporating the real-time syndromic surveillance component is the ability to detect syndromic signals of unknown cause, in real-time, thereby triggering further enhanced epidemiological analyses to determine the underlying cause and aetiology and enable timely public health response. In parallel, this paper raises important questions about MP and points to the need for further work to better understand this pathogen.
